# Unilateral situs inversus of optic disc associated with reduced binocularity and stereoacuity resembling monofixation syndrome

**DOI:** 10.4103/0301-4738.62654

**Published:** 2010

**Authors:** Mihir Kothari, Debapriya N Chatterjee

**Affiliations:** Department of Pediatric Ophthalmology, Aditya Jyot Eye Hospital, Wadala, Mumbai, Maharashtra, India; 1 Department of Strabismus, Aditya Jyot Eye Hospital, Wadala, Mumbai, Maharashtra, India

**Keywords:** Situs inversus, optic disc, monofixation syndrome, anisometropia, congenital optic disc anomaly

## Abstract

Situs inversus of the optic disc is a rare, usually bilateral, congenital embryological abnormality associated with high myopia, optic disc coloboma or tilted optic disc. It is characterized by emergence of the retinal vessels in an anomalous direction with dysversion of the optic disc. In this report we present a 13-year-old boy diagnosed with isolated, unilateral situs inversus of the optic disc associated with reduced binocularity and stereoacuity resembling a monofixation syndrome. The clinicians should be aware of this association and assess the binocularity in patients with unilateral optic disc or macular anomalies. Conversely, patients with reduced binocularity and stereoacuity should be carefully evaluated for macular or optic nerve anomalies, if not associated with strabismus, anisometropia and eccentric fixation. Typical fundus picture, optical coherence tomography and multifocal electro retinogram of the patient would be instructive to a clinician.

Situs inversus of the optic disc is a rare congenital abnormality characterized by emergence of the retinal vessels in an anomalous direction.[1] It is typically seen in myopic patients, patients with the tilted optic disc syndrome and those with the optic pit syndrome.[1–4] It is also reported with optic disc coloboma, Ehlers Danlos syndrome, familial dextrocardia and optic neuritis.[1,5–7] Situs inversus is commonly bilateral and thought to be caused by anomalous insertion of the optic stalk into the optic vesicle resulting in dysversion of the nerve head.

We present a case of unilateral situs inversus in a child associated with mild anisometropia and subnormal binocularity and stereopsis. We believe the characteristic optic disc appearance, optical coherence tomography (OCT) and multifocal electroretinogram (mfERG) would be instructive to the clinicians.

## Case Report

A 13-year-old boy presented with the complaint of diminished vision for far objects in the right eye since one month. There was no significant ocular or medical history. The birth history was normal.

His uncorrected distance visual acuity on Snellen's chart was 20/120 in the right eye and 20/40 in the left eye. His vision improved to 20/20 (Snellen's 6 meter vision chart) in both eyes with −1.5D−1.5D×180° in the right eye and −1.0D−0.5D×170° in the left eye. Detailed ophthalmic examination was significant for suppression of the right eye at six-meter distance on Bagolini's striated glasses.[8] Fusion was present at 40cm. Fusion was also present for near on near Worth four dot test (Cat. 4668, Richmond Products Inc., NM, USA). There was suppression of the right eye beyond 13 feet. The amplitude of fusion was 36 prism diopters (Fusional convergence 26/22 and fusional divergence 10/8). His stereoacuity was 240 seconds of arc on TNO test and 70 seconds of arc on Randot stereo test. Fixation assessment with Lancaster star of a direct ophthalmoscope and with OCT revealed foveal fixation. The cover test demonstrated absence of heterophoria. The 4 prism diopter base out test (Irvine's test) demonstrated absence of bifoveal fixation.

**Figure 1:**
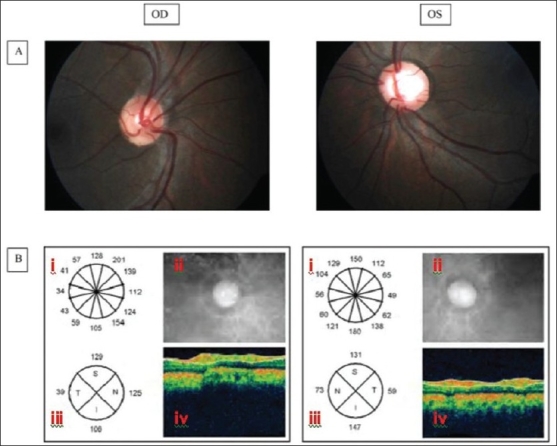
A: 30 degree fundus photograph showing the retinal vessels emerging in nasal direction in the right eye and normal (temporal) direction in the left eye. B: OCT report of the peripapillary Fast RNFL thickness scan (3.4) showing (i) RNFL thickness in clock hour sectors, (ii) fundus image with scan circle, (iii) RNFL thickness of the quadrants and (iv) false color cross-sectional image of the retina. Note the difference in the RNFL thickness between the temporal sector and the nasal sector in the right eye (86 microns) and in the left eye (14 microns)

Fundus examination revealed gross asymmetry in the optic disc cupping and vascular morphology between the two eyes. The right eye had a cup disc ratio of 0.15 with pink neuroretinal rim [Fig. 1A]. The direction of emergence of the retinal vessels was typically nasalward followed by an acute temporal bend. A relatively larger number of nerve fibers appeared to emerge from the supero-nasal area as compared to the supero-temporal and temporal area. There was no glial tissue or peripheral retinal abnormalities that would account for the apparent nasal dragging of the optic disc. This was further substantiated by the measurement of angle kappa (3 degrees in each eye measured by the slit lamp technique). There was no tilting of the optic disc or a peripapillary crescent. The left eye had a normal appearing disc with a cup disc ratio of 0.6. The direction of the emergence of the retinal vessels and nerve fiber distribution appeared normal. Color vision testing with Ishihara's pseudo isochromatic plates and central 30 degree fields tested on Humphrey automated perimeter were normal. Fast retinal nerve fiber layer (RNFL) OCT Scan (3.4) (STRATUS OCT™ Optical Coherence Tomography, Carl Zeiss Meditec, Inc, USA) of the right eye showed abnormal distribution of the RNFL [Fig 1B].

**Figure 2:**
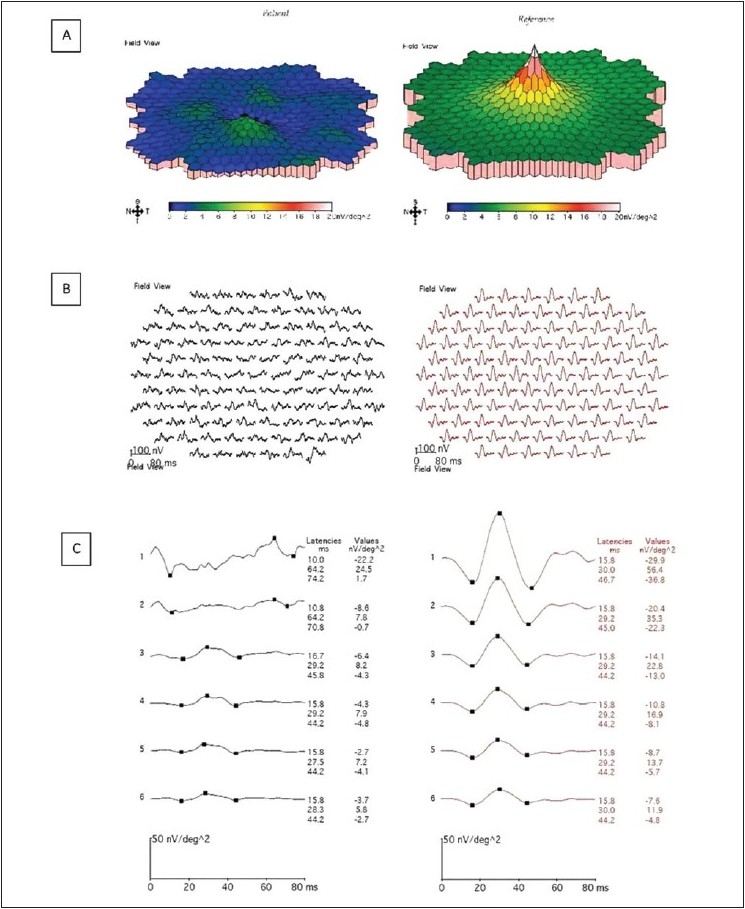
Multifocal electroretinogram (mfERG) output of the right eye showing generalized reduction in the amplitudes of the 1st order kernels. Patient's mfERG responses are on the left side and the reference values are on the right side. A) Color coded 3 dimensional topography map, B) trace arrays and C) averaged responses from the ring 1 (at fovea) to ring 6 (at peripheral macula)

The RNFL thickness in the temporal quadrant measured 86 microns lesser than the nasal quadrant. The left eye appeared normal. A 103 hexagon, mfERG (VERIS system, Electro-Diagnostic Imaging, San Mateo, CA, USA) of the right eye showed delayed latency and generalized depression in the amplitudes of the 1^st^ order kernels [Fig. 2] suggestive of reduced macular function. The left eye was normal. Systemic examination of the child confirmed absence of dextrocardia or systemic situs inversus.

## Discussion

Situs inversus of the optic nerve is characterized by nasalward emergence of the retinal vessels from the optic disc followed by an acute arching of the vessels towards the temporal direction in absence of dragged retina. In this patient it was associated with a 14-fold higher RNFL thickness (86 microns as compared to the normal average of 6 microns, 95% confidence interval 4.5-7.5)[9] in the nasal quadrant compared to the temporal quadrant on the peripapillary OCT scan [Fig 1B], indicative of the nasalization of the retinal nerve fibers due to dysversion of the optic disc.

There was fusion for the near, suppression of the right eye for the distance and reduced stereoacuity on TNO test and Randot test resembling a monofixation syndrome.[10] Although a small anisometropia (1D) was present in this child, other associations of the monofixation syndrome – microtropia or eccentric fixation were absent and that of situs inversus – high myopia, tilted optic disc and optic nerve or retinal coloboma were also absent. This patient had an abnormal mfERG in the right eye associated with situs inversus of optic disc and reduced binocularity and stereopsis. Left eye had normal fundus, OCT, perimetry and mfERG. Neuroimaging or B scan could not be done to rule out coloboma of the optic nerve head or a tilted optic disc syndrome. Similar to this patient, in Park's original paper of 100 monofixators, 19 had primary monofixation syndrome (absence of the above mentioned associations) 40 patients did not have amblyopia and one patient had monofixation syndrome secondary to a macular scar.[10].

In summary, clinicians should be aware of this association and an assessment of binocular fusion and stereoacuity is indicated in patients with unilateral macular or optic nerve abnormality even when the visual acuity is good. Conversely, in patients with reduced binocularity and stereoacuity without anisometropia, microtropia and eccentric fixation, a careful evaluation of macula and optic nerve is warranted.
